# Fellow-Workers and Their Doings

**Published:** 1914-01-24

**Authors:** 


### ROUND THE WORLD.

### Fellow-Workers and their Doings.

[The Editor Invites Early Information and Contributions Marked "Fellow-Workers."]

At Guy's Hospital Sir William Arbuthnot Lane's operating work impresses onlookers with the fact of its remarkable thoroughness. The result is that practitioners obtain many important hints from his clinical teachings that are of the greatest practical use in their routine work. Thus, as a case in point, it does not always occur to medical men that, having made a small incision over, for example, the site of supposed gall-stones or appendicitis, that there is any objection to using such an opening for examining the abdominal cavity in general. The senior surgeon at "Guy's" insists upon recognition of the fact that the use of a keyhole incision for this purpose is to be strongly deprecated, urging the necessity for an opening of such a kind that the whole peritoneal cavity can be thoroughly explored.

Sir Rickman Godlee, Bart., received a popular reception at the University College Medical Society last week, when he met the students on his return from his American tour. The distinguished surgeon, who was given the honorary degree of LL.D. at Toronto, is one of the most entertaining speakers in the medical profession, a circumstance which has helped him in the performance of those social duties which fall to the lot of the President of the Royal College of Surgeons.

London Hospital men will be glad to know that the latest news of Dr. L. A. Smith, who has been on the sick list with appendicitis, is to the effect that he is recovering his strength, and is apparently assured of complete restoration to health and vigour. Just previous to his sudden illness this popular member of the staff at the "London" had been particularly interesting himself in the treatment of cardiac diseases. This is a subject on which he has for many years delivered an annual course of lectures, which have always attracted large audiences of students and practitioners.

Dr. Francis Warner, who has lately retired from active clinical work at the London Hospital, intends to spend a good deal of his time in future at a little place he has taken at Upper Warlingham, near Purley, where he will enjoy the bracing air of the North Downs. Dr. Warner was one of the pioneers in this country in the study of mentally deficient children, and worked very hard in aid of the Mental Deficiency Bill.

Of the four successful candidates for the appointments of medical officer under the National Health Insurance Commission, Dr. Edward William Adams, M.D. Lond., is well known in Sheffield, where he is honorary physician to the Children's Hospital and lecturer in pharmacology and therapeutics at the University. Dr. James Pearse, M.D. Edin., is medical officer of health at Trowbridge, and received his medical education in Edinburgh University. Dr. William Vernon Shaw, M.D. Oxon., has been in practice at Malton (Yorkshire) for some years, where he has been medical officer of health for the district. Dr. Shaw is an Oxford graduate and some years ago was demonstrator of physiology and casualty physician at St. Mary's Hospital. He had a brilliant career as a student, and before private work encroached too much upon his time carried out numerous important physiological and bacteriological researches. Whilst Dr. Barbara Martin Cunningham, who is an M.D., Ch.B. of Edin., and L.M. of the Rotunda Hospital in Dublin, has for some time devoted her attention to public health work.

Dr. Gustave Monod, M.D., Paris, is visiting London in course of a tour on behalf of the French Government in connection with post-graduate instruction. Dr. Monod is a Vichy consultant who has made several communications on gastro-hepatitis and the Vichy regimen to English medical societies and journals. He possesses the distinction of being the only French physician who is an M.R.C.P. Lond.

Dr. Arthur Salusbury MacNalty, M.D. Oxon., who has just been appointed medical inspector of tuberculosis to the Local Government Board, has a good record of scientific work at the London and University College Hospitals and elsewhere. As resident medical officer at the Brompton Hospital for Consumption for two years (1909 to 1911) Dr. MacNalty particularly qualified himself for the branch of public health work he has since joined.

Of the two other medical inspectors now appointed to the tuberculosis department of the Local Government Board, Dr. J. P. Candler, M.D. Cantab., D.P.H, has been working for some years in the pathological department at Claybury Asylum. Since qualifying at Charing Cross Hospital he has devoted much of his time to pathological work, having lately carried out important investigations in connection with the Wassermann reaction, whilst Dr. Frank Seymour, M.D. Dub., D.P.H., has directed the pathological department at the London Temperance Hospital, and has lately held a temporary post similar to that to which he has now been appointed. In his early years at Trinity College, Dublin, Dr. Seymour was awarded a gold medal in experimental science.

Mr. Paul Roth, F.R.C.S., has lately concluded an investigation into a small epidemic of anterior poliomyelitis (infantile paralysis) at Deddington, in Oxfordshire. Mr. Roth, who is the son of Mr. Bernard Roth, the well-known authority on spinal curvature, supports the suggestion that the spread of the disease, in this instance at any rate, was due to the wanderings of the stable-fly, an idea that has gained ground lately, and points out one way at least in which public health authorities may deal with this terrible disease of childhood.

Two very prominent administrators of the Leicester Royal Infirmary have recently celebrated birthdays, Mr. S. F. Stone, the treasurer, receiving the hearty congratulations of the Board on reaching his eightieth anniversary, on the occasion of which he contributed £100 to the Children's Hospital Reconstruction Fund, and Sir Edward Wood, J.P., the chairman, who celebrated his seventy-fifth anniversary on the 16th inst., likewise receiving the hearty felicitations of the governors. Sir Edward Wood has added to his already munificent contributions by a donation of £250 to the Children's Hospital Reconstruction Fund.

